# PDGFRα depletion attenuates glioblastoma stem cells features by modulation of STAT3, RB1 and multiple oncogenic signals

**DOI:** 10.18632/oncotarget.10132

**Published:** 2016-06-17

**Authors:** Carlo Cenciarelli, Hany E. Marei, Armando Felsani, Patrizia Casalbore, Gigliola Sica, Maria Ausiliatrice Puglisi, Angus J.M. Cameron, Alessandro Olivi, Annunziato Mangiola

**Affiliations:** ^1^ Institute of Translational Pharmacology, Department of Biomedical Sciences-National Research Council (IFT-CNR), Rome, Italy; ^2^ Biomedical Research Center, Qatar University, Doha, Qatar; ^3^ Institute of Cell Biology and Neurobiology, Dept. of Biomedical Sciences-National Research Council (IBCN-CNR), Rome, Italy; ^4^ Institute of Histology and Embryology, Catholic University-School of Medicine, Rome, Italy; ^5^ Department of Internal Medicine and Gastroenterology, Agostino Gemelli Hospital, Rome, Italy; ^6^ Barts Cancer Institute, John Vane Science Centre, Queen Mary University of London, London, United Kingdom; ^7^ Institute of Neurosurgery, Department of Head and Neck, Catholic University-School of Medicine, Rome, Italy

**Keywords:** glioblastoma, cancer stem cells, PDGFRα, STAT3, RB1

## Abstract

Platelet derived growth factor receptors (PDGFRs) play an important role in tumor pathogenesis, and they are frequently overexpressed in glioblastoma (GBM). Earlier we have shown a higher protein expression of PDGFR isoforms (α and β) in peritumoral-tissue derived cancer stem cells (p-CSC) than in tumor core (c-CSC) of several GBM affected patients. In the current study, in order to assess the activity of PDGFRα/PDGF-AA signaling axis, we performed time course experiments to monitor the effects of exogenous PDGF-AA on the expression of downstream target genes in c-CSC *vs* p-CSC. Interestingly, in p-CSC we detected the upregulation of Y705-phosphorylated Stat3, concurrent with a decrement of Rb1 protein in its active state, within minutes of PDGF-AA addition. This finding prompted us to elucidate the role of PDGFRα in self-renewal, invasion and differentiation in p-CSC by using short hairpin RNA depletion of PDGFRα expression. Notably, in PDGFRα-depleted cells, protein analysis revealed attenuation of stemness-related and glial markers expression, alongside early activation of the neuronal marker MAP2a/b that correlated with the induction of tumor suppressor Rb1. The *in vitro* reduction of the invasive capacity of PDGFRα-depleted CSC as compared to parental cells correlated with the downmodulation of markers of epithelial-mesenchymal transition phenotype and angiogenesis. Surprisingly, we observed the induction of anti-apoptotic proteins and compensatory oncogenic signals such as EDN1, EDNRB, PRKCB1, PDGF-C and PDGF-D. To conclude, we hypothesize that the newly discovered PDGFRα/Stat3/Rb1 regulatory axis might represent a potential therapeutic target for GBM treatment.

## INTRODUCTION

Among the different types of adult brain tumors, glioblastoma multiforme (GBM) is the most aggressive and angiogenic, but despite the efforts to find effective treatments, these tumors remain incurable [1]. The amplification and/or overexpression of either EGFR or PDGFR can contribute to the malignant phenotype of distinct subsets of human glioblastoma [2]. More recently, a classification of GBM in subclasses has been defined for the selection of the best-tailored therapeutic approaches [3, 4]. Platelet-derived growth factors (PDGFs) isoforms and receptors (PDGFRs) have important functions in the regulation of growth and survival of certain cell types during embryonal development and in control of tissue homeostasis in the adult [5]. A wide range of work from *in vitro* studies to mouse models have implicated the role of the PDGF pathway in cellular invasion and tumor angiogenesis [6]. In fact, over-activity of PDGF signaling is associated with tumor development in brain, prostate, liver, lung, leukemia and colon cancers [7, 8]. Although anti-VEGF treatment has been the major therapeutic target in gliomas, other antiangiogenic agents such as anti-PDGFs or anti-FGFs are currently in preclinical and clinical development [9]. PDGFR includes two receptors (α and β) and four ligands (PDGF-A, PDGF-B, PDGF-C and PDGF-D). The PDGFs bind to the receptors with different affinities. Thus, PDGF-AA, -AB, -BB and -CC induce αα receptor homodimers, PDGF-BB and -DD ββ receptor dimerization, and PDGF-AB, -BB, -CC and -DD αβ receptor dimerization [5]. Ligand-induced dimerization favors autophosphorylation of specific tyrosine residues and subsequent activates downstream signal pathways: PI3K/Akt1/mTOR, Ras/MAPK, PLC-γ/PKC and STAT3. PDGFR binds and activates signal transducers and activator of transcription (STATs). Phosphorylation of Y705 in Stat3 leads to dimerization, nuclear translocation, recognition of Stat3-specific DNA binding elements and up-regulation of various Stat3 downstream target genes, such as Bcl-xl, Bcl-2, Survivin, c-Myc and Cyclin D1. Stat3 regulates tumorigenesis and tumor inflammation and behaves in an oncogenic manner depending on the genetic background of the tumor [1]. In recent studies, Stat3 has been implicated in the self-renewal of neural stem cells and glial differentiation while restricting neuronal differentiation [8–13].

The PKC family consists of fifteen isozymes divided into three subfamilies: conventional (or classical), novel, and atypical. Conventional PKCs contain the isoforms α, βI, βII, and γ. The PDGFR downstream target PKCα plays an important role in migration, tumor growth, angiogenesis and drug resistance in GBM cells [14–16]. In 1992, PKCα was suggested as marker of malignancy for gliomas, and more recently serum PKCα serves as a biomarker for diagnosis of cancers [14, 15]. The invasion/migration of GBM cells induced by TPA, occurs through activation of PKCα/ERK/NF-κB-dependent MMP-9 expression [16]. A positive feedback loop between Wnt5A and phospho-PKC in promotion of epithelial-mesenchymal transition (EMT) in nasopharyngeal carcinoma was disclosed [17]. In addition, PDGF receptors bind to other tyrosine kinase receptors, e.g. EGFR [18]. Retinoblastoma 1 (RB1) gene belongs to a family of three proteins, including also RBL1/p107 and RBL2/p130. Classically the tumor suppressive function of Rb proteins have been mainly attributed to their ability to arrest cell cycle by repressing E2F target genes. When Rb1 is in its active hypophosphorylated state, it represses E2F-mediated transcription by binding, blocks the E2F transactivation domain, and forms complexes with its (DPs transcription factors) partners at cell cycle gene promoters [19]. Conversely, Rb1 phosphorylation initiated by cyclin D-CDK4/6 in response to mitogenic signals, inactivates the Rb1 repressive function by dissociating the Rb1-E2F-DP complexes [19]. The Cancer Genome Atlas Research Network revealed in 2008 that the CycD1-CDK4/6-Rb1 pathway is among the top three most altered pathways in GBM, which makes this an appealing target for cancer therapy [20–22].

We and others recently demonstrated that inhibition of either PDGFRα or PDGFRβ signaling induced apoptosis in glioblastoma stem cells [23, 7]. In the present study, we aimed to assess the effects of PDGFRα depletion on stemness, invasion and differentiation in GBM CSC. Our findings reveal an inverse correlation between Stat3 Y705-phosphorylation and the hypophosphorylated Rb1 instructed by the PDGFRα/PDGF-AA regulatory axis. Further, downmodulation of cell growth, invasion and the EMT phenotype are triggered by PDGFRα depletion in GBM CSC. Surprisingly, we detected the activation of angiogenic and survival pathways as compared to parental cells, which supports a multimodal approach to treat GBM CSC.

## RESULTS

### Activation of PDGFRα/PDGF-AA signaling regulates expression of downstream genes Egr1, Stat3 and Rb1 but not PKCα in GBM CSC

**Figure 1:**
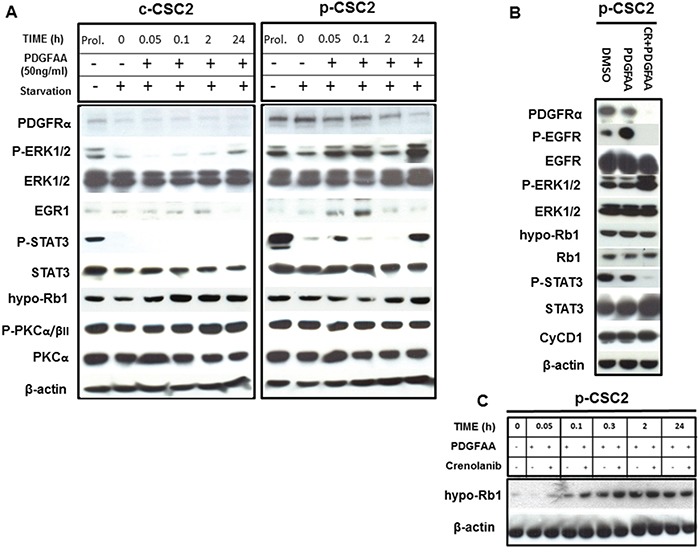
Activation of PDGFRα/PDGF-AA axis induces different modulation of target genes in GBM p-CSC2 than in c-CSC2 **A.** Time course experiment of the effects of PDGF-AA on immediate early genes activation in c-CSC2 *vs* p-CSC2 after 2 days of growth factors starvation. p-CSC2 express Egr1 and Y705-phosphorylated Stat3 after 5 minutes, on the other hand, hypo-Rb1 is significantly downmodulated in p-CSC2 after 10 minutes of treatment. Instead, the levels of T638/641-PKCα/βII remain stable at all time points. **B.** PDGF-AA applied alone or with CR for 24 hours on p-CSC2. Stat3 Y705 phosphorylation levels are impaired by CR treatment, instead hypo-Rb1 and total Rb1 protein expression are not affected after 24 hours by PDGFR inhibition. Cyclin D1 protein levels are not modulated in the same conditions. β-actin is used as control of equal proteins loading. **C.** Time course experiment to evaluate the effects of PDGF-AA alone or combined with CR on the regulation of hypo-Rb1 in p-CSC2. CR upregulates hypo-Rb1 expression respect to PDGF-AA alone.

Cancer stem cells from GBM were isolated as described previously [23, 24]. We were able to collect either core- (c-CSC) or peritumor tissue-derived cancer stem cells (p-CSC) from several primary GBM samples; the two types of CSC had quite different tumorigenic potential and exclusive genetic anomalies [23, 24]. To demonstrate that the high expression of PDGFRα in p-CSC2 [23], confers a higher PDGF response than in c-CSC2, both cell types, were growth factors starved and then, exposed to exogenous PDGF-AA (50 ng/ml) over a time course. In these experimental conditions (Figure 1A), we found more rapid activation of p-Erk1/2 in p-CSC2 than in c-CSC2 as reported previously [23]. Moreover, Egr1 (Early growth response 1) protein and Stat3 Y705 phosphorylation were induced as early as 5 minutes after PDGF-AA stimulation (Figure 1A). Strikingly, we noted a tight correlation between this phosphorylation event and the transient decrement of the tumor suppressor Rb1 in its active state, that occurred within 10 minutes, as detected by a monoclonal antibody that specifically recognizes hypophosphorylated active Rb1 (hypo-Rb1). A densitometric analysis (data not shown) showed a 70% signal reduction at this time point compared to time point zero, followed by a gradual recovery. The engagement of PDGFRα with its ligand induced the gradual disappearance of the receptor 2 hours after PDGF-AA stimulus. The high expression and phosphorylation of PKCα/βII isoforms was not altered by PDGF-AA stimulation in either cell populations (Figure 1A).

### Block of PDGFRα activity by Crenolanib reduces the phosphorylation of Rb1 and Stat3 Y705

To assess whether the decrement of hypo-Rb1 was PDGFRα/PDGF-AA axis-dependent, a time course experiment was performed to evaluate the effects of PDGF-AA applied alone or in combination with Crenolanib (CR, a potent inhibitor of PDGFR signaling), on p-CSC2 two days after growth factors withdrawal. Rb1 phosphorylation was monitored at short time intervals (5-10-30-120 minutes) up to 24 after the start of PDGF-AA stimulation. CR-treated cells showed upregulation of the hypo-Rb1 between 5 and 30 minute after the treatment as compared to PDGF-AA alone. A densitometric analysis (data not shown) showed an increase of 50% of hypo-Rb1 signal within the first half hour in CR-treated cells compared to PDGF-AA alone. Two hours after the treatments the levels of the hypo-Rb1 return to baseline (Figure 1C). In contrast, Stat3 Y705 phosphorylation remained silenced at 24 hours by CR treatment (Figure 1B). Previous studies showed that the epidermal growth factor receptor (EGFR) could be transactivated by PDGFs stimulation and that EGFR transactivation was required for PDGF-stimulated cell migration [18]. In corroboration, we showed that PDGF-AA induced EGFR Y1068-phosphorylation, and EGFR transactivation was inhibited by Crenolanib treatment (Figure 1B). CR was also found to upregulate Erk1/2 downstream of both the PDGFR and the EGFR (Figure 1B).

### shPDGFRα-GBM CSC display changes in cell morphology and EMT phenotypes, which correlate with modifications in the molecular profile

**Figure 2:**
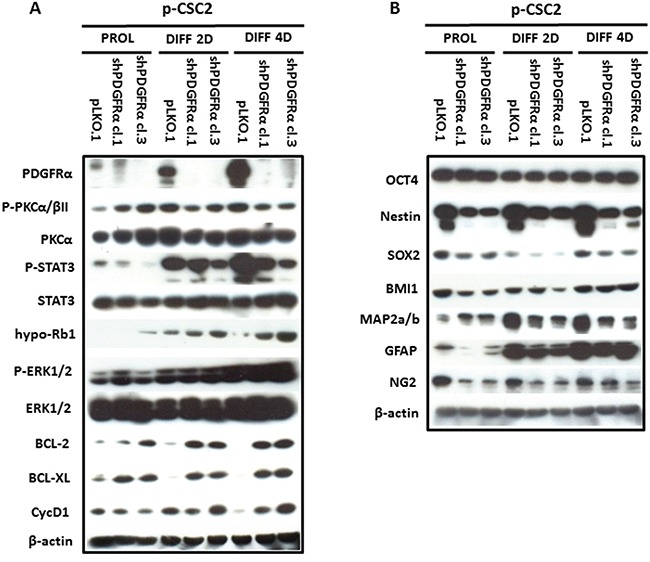
Modulation of multipotency, differentiation and survival markers in PDGFRα-depleted GBM CSC vs control cells **A.** Western blot analysis reveals that depletion of PDGFRα in p-CSC2 induces the downmodulation of Stat3 Y705 phosphorylation in both proliferating cell clones, instead their levels raised up in differentiation conditions. The hypo-Rb1 levels result overexpressed in clone 3 in all conditions. Instead the clone 1, shows an increase of hypo-Rb1 at 2 and 4 day of differentiation with respect to pLKO.1 cells. In parallel, Bcl-2 and Bcl-xL proteins and Erk1/2 pathway were upregulated in both cell clones in comparison with pLKO.1 cells in differentiaton conditions. T638/641 phosphorylated-PKCα/βII isoforms expression levels are slightly induced in proliferating PDGFRα-depleted cell clones, but negatively modulated in differentiated ones than pLKO.1 cells. **B.** The downmodulation of multipotent protein markers (Nestin, Sox2, Bmi1), except for Oct4, is accompanied with early induction of the neuronal marker MAP2a/b in proliferating PDGFRα-depleted cell clones vs pLKO.1. Similar results were observed during cell differentiation, except for MAP2a/b that increases in pLKO.1 with respect to PDGFRα-depleted cells.

**Figure 3:**
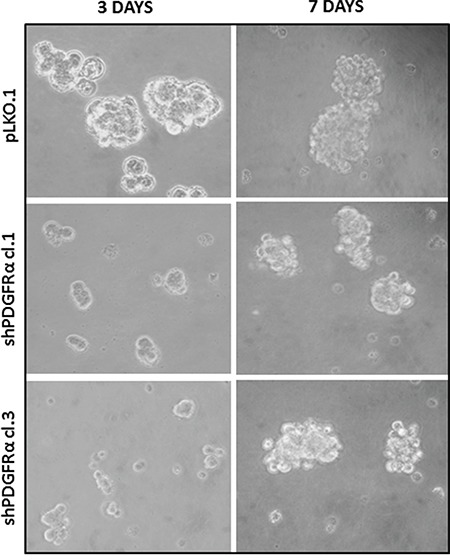
Neurospheres formation impairment of PDGFRα-depleted GBM CSC *vs* control cells Microscopic analysis at 3 and 7 days in growth conditions reveals a diminished ability of p-CSC2 depleted of PDGFRα to make neurospheres *in vitro* compared to control cells (pLKO.1) over the entire course of the experiment.

**Figure 4:**
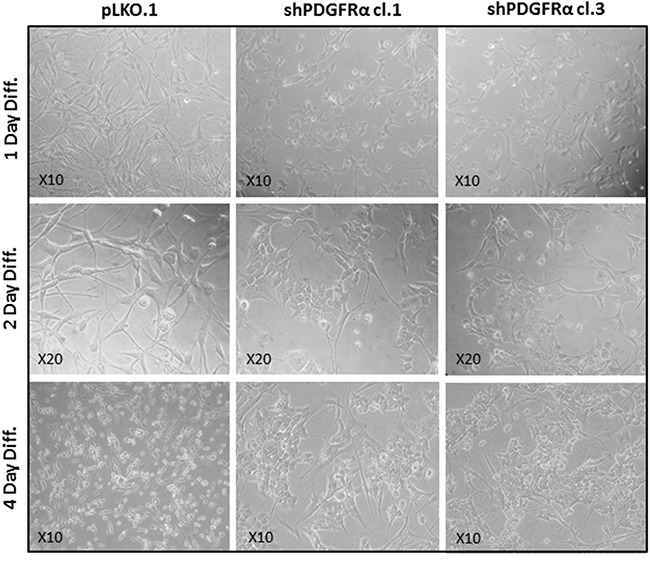
PDGFRα-depleted GBM CSC show phenotypic changes *vs* control cells Phase-contrast images captured after 1-2-4 days in differentiation conditions revealed morphological changes between shPDGFRα-CSC clone 1 and clone 3 in comparison with pLKO.1. The aspect of pLKO.1 cells is more similar to the mesenchymal phenotype with a flat cell morphology. Conversely, the shPDGFR-CSC cell clones displayed an epithelial appearance with crowded cell clusters among few elongated cells, which is clear manifested at 4 days of the cell differentiation.

**Figure 5:**
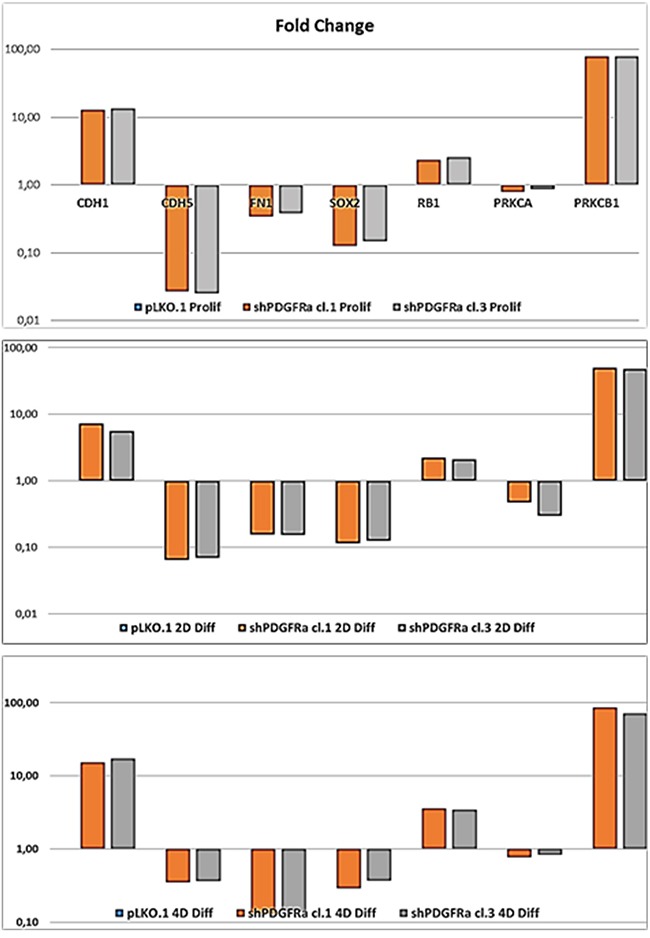
PDGFRα deletion induces different expression of target genes in GBM CSC in proliferation and differentiation conditions RT-qPCR analysis shows a significant modulation of gene expression of cell-cell (CDH1, CDH5) and cell-substrate (FN1) molecules interactions, the stem cell marker SOX2 and oncogenic signaling molecules (PRKCA, PRKCB1). Progressive upregulation of the tumor suppressor RB1 and PRKCB1 isoform during cell differentiation is shown. Results represent the mean of two independent experiments performed in triplicate. Fold change (Fc).

To demonstrate the central role of PDGFRα in CSC proliferation and differentiation, we interfered with PDGFRα gene expression using short hairpin RNA (shRNA) sequences transduced by a lentivirus-based system. We selected two PDGFRα-directed shRNA clones (1 and 3) of p-CSC2 that displayed a robust silencing of PDGFRα compared to transduced control cells (pLKO.1) (Figure 2A). Depletion of PDGFRα expression was associated with a reduction in cell growth as reported previously [23], and with a concomitant impairment of neurosphere formation ability, consistently observed over the entire seven days of time course (Figure 3). The two cell clones showed evident morphological changes if induced to differentiate in presence of 5% FCS. After 2 and 4 days in differentiation conditions, shPDGFRα-CSC showed an epithelial cell morphology, with formation of adherent cell clusters and few cells with an elongated phenotype. In contrast, the pLKO.1 control cells showed a flat morphology with a clear mesenchymal phenotype, evenly distributed on the plate (Figure 4). These observations prompted us to explore the gene expression of epithelial cell junction proteins such as E-cadherin (CDH1) and VE-Cadherin (CDH5) or ECM molecules such as fibronectin (FN1) by a RT-qPCR analysis. CDH1 mRNA expression was monitored in PDGFRα-depleted CSCs during cell proliferation. Both clone 1 and 3 showed higher expression compared with control cells, with a fold change of 13.1 and 13.6 respectively in proliferating cells and with a peak of activation at 15.2 and 17.3 Fc after 4 days of differentiation (Figure 5). Our finding were consistent with that published in various cell lines, reporting an inverse correlation between CDH1 expression and invasiveness [25]. CDH5 mRNA levels were drastically downmodulated in proliferating cell clones and reached 0.35 and 0.36 Fc in 4 days-differentiated cells respectively (Figure 5). Similarly, FN1 mRNA expression was diminished to 0.34 and 0.38 fold in proliferating shPDGFRα-CSC clones compared to pLKO.1, and went down to 0.16 and 0.15 Fc in 2 days-differentiated cells. Similar results were seen at 4 days (Figure 5). As reported by the literature, the combined loss of CDH1, and the gain of mesenchymal markers, such as vimentin, FN1 and N-cadherin favor the EMT program [26].

### PDGFRα depletion downmodulates stemness-related and glial markers, induces early expression of neuronal marker and RB1 but does not affect PKCα in GBM p-CSC

To assess the role of PDGFRα in the regulation of stemness of GBM CSC, we carried out Western blots and RT-qPCR to elucidate the effects of a diminished PDGFRα expression on stemness-related genes. We observed a decrease in protein expression for Nestin, Sox2, Bmi1, but no modulation of Oct4 (Figure 2B). RT-qPCR analysis confirmed the decrease of SOX2 mRNA to 0.11 and 0.13 fold in clone 1 and 3 compared to control cells. Similar results were confirmed at 2 and 4 days-differentiated p-CSC2 (Figure 5). Attenuation of stemness in shPDGFRα-CSC clones correlated with early induction of late neuronal differentiation marker MAP2a/b protein in proliferating cells (Figure 2B), also monitored as mRNA (data not shown). Genetic and pharmacological depletion of PDGFRα triggered the activation of tumor suppressor RB1 gene, a key factor controlling cell cycle exit and differentiation in several cell models. As shown in Figure 5, PDGFRα-depleted CSC clone 1 and 3 displayed the induction of RB1 mRNA up to 2.35 and 2.59 fold respectively compared to control cells. Expression levels raised a peak to 3.59 and 3.45 Fc in 4 days-differentiated cells respectively. Western blot analysis revealed the increase of hypo-Rb1 in proliferating clone 3, or in both differentiated clones compared to pLKO.1 (Figure 2A). Under the same culture conditions, we noticed a decrease of Stat3 Y705-phosphorylation in both clones in comparison to pLKO.1 cells (Figure 2A). Conversely, we reported a diminished expression of glial fibrillary acidic protein (GFAP) and the chondroitin sulfate proteoglycan NG2 (Figure 2B). The latter is probably the result of lack of a physical association with PDGFRα, which is an obstacle to the formation of new oligodendroglial precursors NG2+ve [28]. The reduced PDGFRα expression correlated with higher levels of Bcl-2 and Bcl-xL compared to control cells, suggesting a potential role of these genes in cell survival in either proliferating or differentiated cells. These results paralleled with an increase in CycD1 protein in differentiated cells and a slight decrease in proliferating cells. The latter effect likely correlated to cell proliferation impairment induced by PDGFRα deletion. Because of the critical role of PKCα in promoting EMT phenotype and malignant progression of glioblastoma cells, we hypothesized that PKCα could regulate stemness of GBM CSC downstream of the PDGFRα signaling cascade. To answer the question, we performed shRNA targeting of PKCα expression in GBM p-CSC2. The results revealed no variation of the multipotent stem cells markers Nestin or Sox2, even with the addition of PDGF-AA for three days (Figure S1). This finding reveals that PKCα is probably not involved in regulation of stemness through the PDGFRα signaling.

### PDGFRα depletion inhibits the invasiveness of GBM CSC and triggers EDN1 signaling, PDGF-C and PDGF-D expression

**Figure 6:**
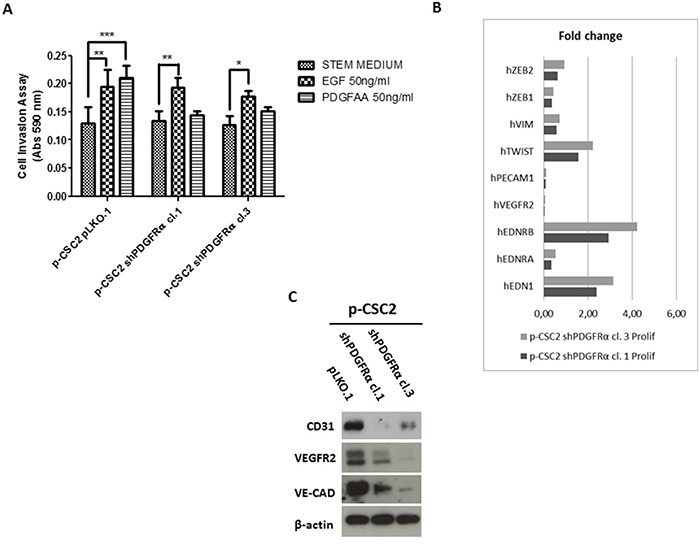
shPDGFRα-CSC clones display a significant reduction of invasiveness and angiogenesis compared to pLKO.1 **A.** In the absence of growth factors (Stem Medium) all cells display the same invasive capacity. Addition of EGF promoted invasiveness in both cell clone 1 and 3 *vs* pLKO.1 cells. PDGF-AA addition significantly promoted pLKO.1 cells invasion but does not affect p-CSC2 shPDGFRα cell clones invasiveness. **B.** RT-qPCR analysis of key genes for angiogenesis and EMT. PECAM1, VEGFR2 are dramatically repressed in p-CSC2 shPDGFRα clone 1 and 3 with respect to pLKO.1. EDN1 and its receptor EDNRB are upregulated in PDGFRα-depleted cells compared to pLKO.1. The main EMT markers such as ZEB1, ZEB2 and VIM, except for TWIST, are significantly downmodulated in PDGFRα-depleted cells compared to pLKO.1 cells. Error bars represent the mean ± SD of two independent experiments performed in triplicate. *, P<0.05, **, P<0.01**, ***, P<0.001 *vs*. control. **C.** Western blot analysis for endothelial markers in p-CSC2 shPDGFRα clone 1 and 3 with regard to pLKO.1. A significant decrease is reported for CD31, VEGFR2 and VE-cadherin proteins in PDGFRα-depleted cell vs control cells.

**Figure 7:**
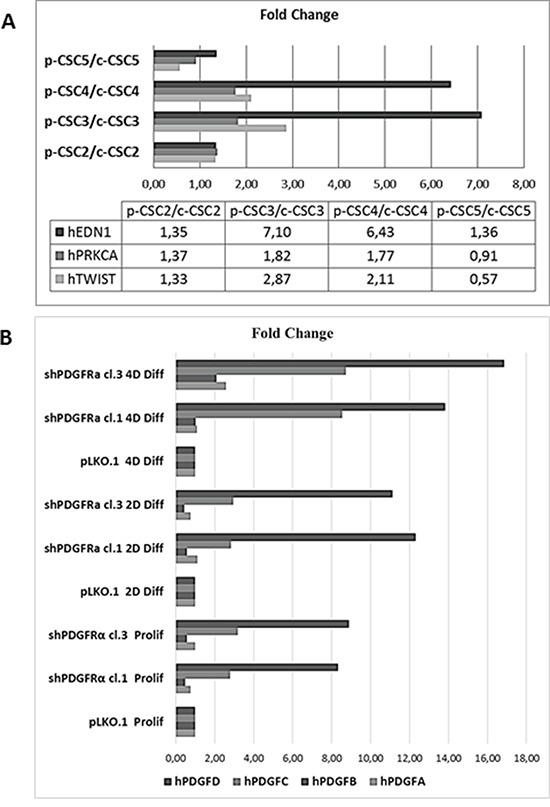
RT-qPCR analysis of EMT markers in several GBM CSCs and of PDGF isoforms profile in pCSC2 shPDGFRα *vs* pLKO.1 **A.** We compared several GBM p-CSCs *vs* the respective GBM c-CSCs for EDN1, PRKCA and TWIST mRNA expression. **B.** mRNA expression profile of PDGF isoforms highlights the elevated induction of PDGF-C and PDGF-D in p-CSC2 shPDGFRα vs pLKO.1 either in proliferating or differentiated cells. Results represent the mean of two independent experiments performed in triplicate.

Formerly we reported that GBM p-CSC2 displayed higher invasive capacity through Matrigel with respect to the counterpart c-CSC2 [23]. Herein, we aim to demonstrate the role of PDGFRα/PDGF-AA axis in the regulation of the invasive capacity of p-CSC2. Using an *in vitro* cell invasion assay we demonstrated a significant reduction in PDGF-AA stimulated invasion of shPDGFRα-CSC clones compared to pLKO.1. Demonstrating specificity, no difference in the invasive capacity was observed using EGF as chemoattractant (Figure 6A). The EMT leads to a reduced cellular adhesion, changes in cytoskeletal organization and acquired potential for cell migration, all this process is associated to changes in expression of transcription factors [30]. To corroborate the role of PDGFRα on the invasive phenotype of p-CSC2, we completed the RT-qPCR analysis of critical genes for EMT such as ZEB1, ZEB2, TWIST1 and Vimentin (VIM). Our results displayed a significant decrease of VIM, ZEB1, ZEB2, but not TWIST1 in both proliferating cell clones (Figure 6B). Similarly, we noticed a decrease of angiogenic markers (Figure 6B) such as VEGFR2, PECAM1 (CD31), and CDH5 as shown previously (Figure 5). In parallel, Western blot analysis display a significant decrease of CD31, VEGFR2 and VE-Cadherin proteins, corroborating mRNA expression levels (Figure 6C). Unexpectedly, we noticed an increase of mRNA expression for endothelin1 (EDN1), the receptor EDNRB (Figure 6B), its target gene PRKCB1, but not PRKCA (Figure 5). In Figure 5 we reported a significant upregulation of PRKCB1 gene expression in proliferating (79.4 and 78.9 Fc respectively) and differentiated PDGFRα-depleted cell clones 1 and 3 (86.3 and 71.8 Fc respectively) compared with pLKO.1. Instead, PRKCA gene expression was slightly reduced either in proliferating (0.78 and 0.86 Fc respectively) or differentiated PDGFRα-depleted cell clone 1 and 3 (0.7 and 0.8 Fc respectively). Consistently, PKCα protein expression and phosphorylation of PKCα/βII isoforms are reduced in differentiation conditions in cell clones compared to pLKO.1 (Figure 2A). In as much as peritumor tissue-derived CSC presented greater chemoresistance to treatments respect to core tumor derived-CSC [23], we decided to investigate by RT-qPCR the expression of the main modulators of EMT and/or drug resistance such as TWIST, EDN1 and PRKCA in several samples of GBM CSC (Figure 7B). We observed a slight increase of PRKCA in p-CSC compared to respective c-CSC pools, except for CSC5. EDN1 and TWIST expression levels were upregulated several fold in the majority of p-CSC in comparison to c-CSC (Figure 7A). We examined the mRNA expression profile of PDGF ligand isoforms in PDGFRα-depleted cells. Interestingly, we noticed that PDGF-C and PDGF–D were highly expressed in proliferating PDGFRα-depleted CSC as compared to pLKO.1 cells. PDGF-C reached 2.8 and 3.2 Fc in proliferating clone1 and 3 respectively, while PDGF-D reached a peak of 8.3 and 8.9 Fc respectively. At 2 and 4 days-differentiated cells these values were significantly higher: PDGF-C reached a peak at day 4 with 8.5 and 8.7 Fc in clone 1 and 3 respectively, instead PDGF-D raised up to 13.8 and 16.8 Fc respectively as compared with pLKO.1 (Figure 7B).

## DISCUSSION

Our work aimed to assess the role of PDGFRα signaling in self-renewal, differentiation, invasion and EMT phenotype in glioblastoma CSC through the knockdown of PDGFRα expression. Previous studies showed that either genetic or pharmacological targeting of PDGFRβ or PDGFRα inhibited self-renewal, survival, tumor growth and invasiveness of GBM CSC [7, 23]. Overexpression of PDGFRα in p-CSC of six GBM cases published previously by our group prompted us to make the hypothesis that peritumor tissue-derived GBM CSC might be more responsive to inhibition of PDGFR activity [23]. In fact, we have shown that a highly potent inhibitor of PDGFR activity, Crenolanib, induced apoptosis in several GBM CSC (p-CSC and c-CSC) which increased in combination with the inhibition of EGFR activity [23].

In order to understand the meaning of PDGFRα overexpression in GBM p-CSC2, we tested the effects of PDGF-AA stimulation on expression of canonical target pathways such as Erk1/2, Stat3, Egr1. Transient Stat3 activation occurred at early time points and, it was subsequently turned off. At later time points, Stat3 activation was once more increased. This latter effect is most likely indirect and it is mediated by progrowth signals triggered by PDGFRα/PDGF-AA positive loop. In the same experiment, we observed a transient downmodulation of hypo-Rb1, which represents inactivation of the tumor suppressive function of Rb1 downstream of the PDGFRα/PDGFAA axis. The subsequent recovery of hypo-Rb1 expression correlated with a decrease in PDGFRα expression. The block of PDGFRα activity by Crenolanib also increased the hypo-Rb1 levels with respect to PDGF-AA alone, and returned to steady state levels at later time points. This observation suggests that Rb1 and PDGFR signaling are interconnected to control cell fate, but more experiments are warranted to elucidate the molecular mechanisms of this relationship.

The induction of the transcription factor Egr1, a member of a zinc-finger transcription factor family, goes in the direction of an oncogenic regulatory loop directed by PDGF-AA. Accordingly Sakakini et al. reported that the nuclear expression of Egr1 is restricted to proliferating cells in high-grade gliomas, and in primary cultures of glioma stem-like cells, Egr1 contributes to stemness marker expression and proliferation by orchestrating a PDGF-AA-dependent growth stimulatory loop [28].

**Figure 8:**
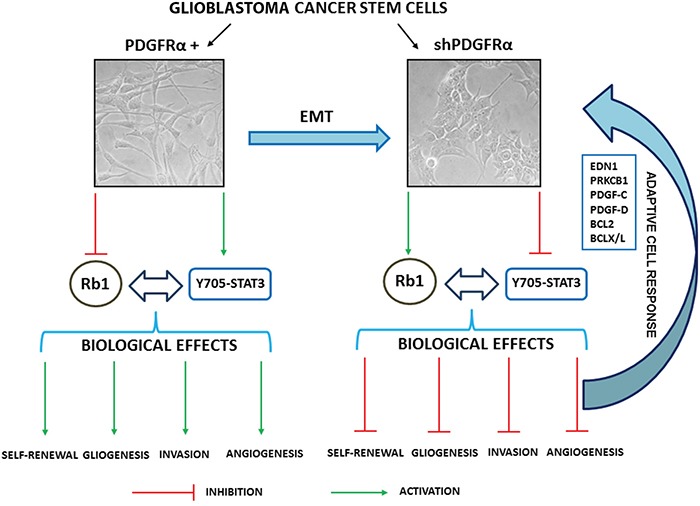
Schematic representation of the biological and molecular effects triggered by depletion of PDGFRα in GBM p-CSC2

A prominent effect induced by PDGFRα depletion in GBM CSC was cell growth impairment and an associated reduction in ability of GBM CSC to make neurospheres *in vitro*. PDGFRα deletion also contributed to a reduced expression of stemness-associated genes as well as the downmodulation of EMT and angiogenic markers. A schematic representation of biological and molecular effects triggered by silencing of PDGFRα in GBM CSC is shown (Figure 8). EMT is a physiological process occuring during embryogenesis that appears to be reinstated under certain pathological conditions, such as cancer. In the EMT process, epithelial cells lose apico-basal polarity and gain properties of mesenchymal cells, and become motile and invasive [29–31].

PDGFRα-depleted CSC revealed the concomitant upregulation of angiogenic and survival pathways apparently in contrast with the downmodulation of endothelial markers such as VEGFR2, PECAM1 and VE-Cadherin. We hypothesize that GBM CSC counteracted angiogenic inhibitory signals by activation of progrowth and prosurvival signals, which comprise PDGF-C, PDGF-D, EDNRB/EDN1/PRKCB1, Bcl-2/Bcl-xL and extracellular signal-regulated kinase (Erk1/2) signaling pathways [8, 32–36]. Several studies have demonstrated that PDGF-C and PDGF-D expression were ubiquitous in brain tumor cells and tissues but were very low or absent in normal adult and fetal brain [35]. High levels of those soluble factors in glioma specimens are associated with more rapid disease recurrence and poorer overall survival [35]. PDGF-C has been reported to induce angiogenic activity indirectly, via upregulation of VEGF and directly, via activation of PDGFRα. In particular, PDGF-C plays an important role in glioma vessel maturation and permeability, and can attenuate the response and escape from anti-VEGF therapy [35]. *In vitro* studies reported that inhibition of Bcl-2/Bcl-xL, along with an antitumor agent that induces TRAIL pathway-mediated cell death give a strong synergistic anti-proliferative effect on pediatric, adult, proneural GBM and glioma stem-like cells [36]. Our data support the existence of autocrine and paracrine signals and implicate for a role of PDGF-C, -D and EDN1 signaling in GBM tumor growth. Liu and colleagues reported that EDN3/EDNRB signaling is involved in maintaining GBM CSC migration, stemness and survival [33].

Dong et al. reported that the selective inhibition of PDGFR by the Imatinib, a drug analog of Crenolanib, elicited the activation of Erk1/2 in malignant glioma cells. The activation of Erk1/2 induced by the Imatinib treatment was related to the S-phase re-entry of the cell cycle in one of the three glioma cells tested [38]. Evenly the up regulation of CycD1 observed in PDGFRα-depleted cells compared to pLKO.1, particularly evident at 4th day of differentiation, would agree with the presence of autocrine and paracrine mitogenic stimuli as argued previously. The overall results reinforce the rationale of using combined anti-angiogenic and multiple approaches to improve the therapeutic response for GBM [39, 35, 9].

Tam and colleagues reported that PKCα overexpression upregulates AP1, which in turn mediates Notch4 activity [40]. Activated Notch4 is closely associated with the promotion of estrogen-independence and chemotherapy resistance in breast cancer cells. In different cell contexts such as intestinal, pancreatic and mammary cells, PKCα has anti-proliferative effects [41]. PKCα is a downstream target of PDGFR signaling and despite the constitutive phosphorylation on T638-641, it does not appear to be modulated at transcriptional level in proliferation and differentiation conditions. Nevertheless, we hypothesized that PKCα could be involved in stemness regulation in GBM CSC, but unexpectedly we noticed no significant modulation of the multipotency stem cell markers Nestin and Sox2 in PKCα-depleted CSC as compared to parental cells. Further experiments needed to clarify the role of PKCα in the maintenance, invasiveness and differentiation of GBM CSC. Our data led us to assume a compensatory mechanism as described formerly because of the increase of PRKCB1 gene expression, however this result is not consistent with the levels of T638-641 phosphorylation of PKCβII isoform and we think that the issue should be addressed in details. For PKCβ isoforms there are conflicting reports, as some indicate PKCβ expression, while others not [42]. Clinical studies have evaluated the acceptable tolerability of enzastaurin (an inhibitor of PKCβ and PI3K/AKT) in patients with recurrent GBM, but the authors reported that it did not have a superior efficacy of the alkylating agent Lomustine [43, 44]. However, there is a paucity of data on the role of PKCβ isoforms in CSC and further experiments are required to reveal their role in glioma pathogenesis.

We have reported the up regulation of RB1 at transcriptional and protein level in its active state in PDGFRα-depleted CSC either in proliferating or differentiated CSC, which correlated with Stat3 activity decline. Our data reported for the first time that the PDGFRα/PDGF-AA axis is a regulator of the crosstalk between Stat3 and Rb1 signals. In the current study, Stat3 seems to play a dual role as oncogene and tumor suppressor in GBM CSC. Evidences from the literature, report Stat3 persistently phosphorylated and associated with an unfavorable prognosis in GBM. In the current study, the markers of neural stem cell multipotency decrease as much as Stat3 activity decreased upon PDGFRα inhibition, suggesting that Stat3 is required for maintenance of the stem-like features of these cells [1, 13]. Furthermore, JAK-Stat signaling pathway selectively enhanced differentiation of neural stem cells towards a glial lineage and inhibited differentiation of neural precursors along a neuronal lineage [11, 12]. Here we show that the upregulation of Stat3 activity during GBM CSC differentiation is consistent with higher levels of GFAP with respect to PDGFRα-depleted cells, which instead revealed a decrease of GFAP and NG2 expression and the early activation of neuronal marker MAP2a/b in proliferating cells [27]. Besides a role of PDGFRα in glial differentiation, the block of PDGFRα activity *in vitro* promoted the reduction of NG2 expression and tumor cells growth as reported by Pilkington [45]. Accordingly Chekenya et al. described that the grafting of NG2+ve GBM cell lines displayed an *in vivo* growth advantage, which was associated with tumor angiogenesis [46].

The Rb1 pathway is altered in almost 70% of human cancer types and this pathway is mutated in 78% of cases of GBM. Ichimura et al. reported that among 120 GBM, 40% had no wild-type CDKN2A gene, 12% amplified the CDK4 gene, and 14% had no wild-type RB1 gene [47], and the majority of the GMB (64%) had only one of these abnormalities [48]. Nakamura et al. reported that 93% of tumors with Rb1 expression had a normal RB1 gene status [49]. In our paper, we reported higher expression of RB1 gene transcription and hypophosphorylated Rb1 protein in PDGFRα-depleted GBM CSC compared to control cells, thus on the basis of the literature, we could assume that RB1 gene most likely has a normal status. On the other hand, we could not rule out CDKN2A and CDK4 gene alterations. In addition, the complexity of Rb1 pathway is often accompanied by gain-of-function of p53, resulting in dysregulation of tightly regulated cellular processes such as cell cycle, upregulation of survival and oncogenic signals as observed in our PDGFRα-depleted GBM CSC [50]. Several preclinical and clinical studies aimed to inhibit the CDK4/CDK6 kinase activity as components that lead to Rb1 inactivation by hyperphosphorylation, and consequent suppression of cell cycle arrest [51–53]. We assumed that the Rb1 gene activation detected in PDGFRα-depleted GBM CSC clones would promote anti-proliferative and anti-apoptotic effects and along with Stat3 might induce the molecular profile change observed in differentiated PDGFRα-depleted GBM CSC. Rb1 is also involved in other cellular processes such as the induction of terminal cellular differentiation, maintenance of genetic stability, protection from apoptotic insults in GBM and cancer stem cells [19, 48, 50–53].

After all we found that the inhibition of a single molecular target by a genetic approach or the application of receptor tyrosine kinase inhibitor as a single drug could provoke adaptive effects in tumor cells such as the activation of compensatory oncogenic signals [38]. Therefore, combination therapies could be a better way to kill tumor stem cells. As an example, Ziegler et al., demonstrated that the inhibition of PDGFR in human glioblastoma cells, is counteracted by inhibitor of apoptosis proteins (IAP), and concomitant inhibition of PDGFR with inactivation of IAPs resulted in increased apoptosis [38, 49].

Further experiments should be addressed to unveil the molecular mechanisms underlying the molecular crosstalk between Rb1 and Stat3 pathways directed by PDGFRα/PDGFAA axis in GBM CSC. In fact, clinical trials are ongoing to target PDGFR amplification or Stat3 activity in patients with malignant gliomas. Unfortunately, the results of these clinical trials are not yet available, so it is early to draw conclusions. We hypothesize that the newly discovered PDGFRα/Stat3/Rb1 regulatory axis might represent an appealing therapeutic target for GBM treatment.

## MATERIALS AND METHODS

### Ethical statement

Procedures for collection of adult human GBM CSC were approved by the Ethical Committee of the Catholic University of Rome as reported previously [24, 25]. The informed consent was obtained and all patients were fully aware of the aims and scope of this work. The ethical principles of the declaration of Helsinki, were strictly followed [24, 25].

### Cell culture of human glioblastoma cancer stem cells

We have used the same clinical materials reported in our previous papers [24]. In brief, the CSC cells were retrieved from adult patients affected by GBM and undergoing craniotomy at the Institute of Neurosurgery, Catholic University-School of Medicine of Rome, Italy. Dissociated cells were cultured in proliferation medium containing human recombinant EGF (20 ng/ml; PeproTech, Rocky Hill, NJ), human recombinant bFGF (10 ng/ml; PeproTech), in DMEM/F12 (1:1) serum-free medium (Invitrogen, Carlsband, CA) as reported previously [24]. Floating neurospheres were dissociated with Accutase at 37°C (Merck-Millipore). Neurospheres culture were passaged up to passage P30 and the experiments were performed between P20 and P30.

### shRNA, transfection and lentivirus production

Experiments of RNA interference are carried out using Mission Lentivirus-based shRNA for PDGFRα (NM_006206-Sigma-Adrich) and PKCα (NM_002737.2-Sigma-Aldrich) as reported previously [24]. We selected several puromycin resistant PDGFRα-directed shRNA cell clones (shPDGFRα-CSC cl.1, 3), but only two significantly targeted PDGFRα expression (TRCN0000195132/clone 1 and TRCN0000196928/clone 3. Similarly, we selected GBM CSC clones expressing shRNA for PKCα (TRCN0000233511 and TRCN0000233513).

### Western blots

GBM CSC are seeded as single cells (1x10^6^/p90 dish) in proliferation medium, and collected 2-3 days later for Western blot analysis. Afterwards, cells were collected and washed with PBS containing proteases inhibitors before protein extraction in 100-150μl of lysis buffer (1% NP-40, 0.01% SDS, 20 mM Tris–HCl pH 7.4, 300 mM NaCl, 1 mM EDTA, 1 mM Na3VO4 and protease inhibitors cocktail from Sigma–Aldrich). Then, cells were sonicated with two pulses of 5 sec at 50% of amplitude (Sonics and Materials, Newtown, CT). Equal amounts (30μg/lane) of total protein extracts, quantified by Bio-Rad protein Assay (Bio-Rad, Munchen, Germany), were loaded on PAGE Bis-Tris gels (FISHER), and transferred on Hybond-P Extra membrane (Amersham Biosciences, GE Healthcare Life Science-Buckinghamshire, UK). Filters were immunoblotted using the following primary antibodies: rabbit anti-EGFR, rabbit anti-Y1068-EGFR, goat anti-PDGFRα, rabbit anti-T202/Y204-ERK1/2 and anti-ERK1/2, rabbit anti-Y705-STAT3 and rabbit anti-STAT3, rabbit anti-T638/641-PKCα/βII, rabbit anti-PCKα, rabbit anti-VEGFR2 (all purchased from Cell Signaling, MA-USA), mouse anti-underphosphorylated Rb1 (BD Pharmigen), Rabbit anti-Rb1 (Cell Signaling), mouse anti-BCL-2 (Dako), mouse anti-Nestin (Millipore), mouse anti-GFAP (Covance), rabbit anti-NG2 (Chemicon), mouse anti-MAP2a/b and rabbit anti-OCT4 (Millipore), rabbit anti-BCL-x/L, rabbit anti-CycD1, rabbit anti-SOX2, rabbit anti-BMI1 and rabbit anti-EGR1, rabbit anti-CD31, goat anti-VE-Cadherin (Santa Cruz-USA), mouse anti-β-actin (SIGMA). After three washing with TBS-Tween buffer, immuno-reactive proteins were detected using rabbit anti-mouse, donkey-anti-rabbit and donkey anti-goat horseradish peroxidase-conjugated secondary antibodies directed to the appropriate primary antibodies (Jackson Immunoresearch Laboratories, West Grove, PA). The proteins were then visualized using the chemiluminescence system (Millipore). Gels and images acquisition were done by HP Photosmart Essential Ver. 1.12 and Adobe Photoshop CS5 respectively. The densitometric analysis of protein bands normalized against to β-actin protein levels was performed from two independent experiments using the ImageJ software (NIH, USA).

### Cell proliferation, differentiation and pharmacological treatment of GBM CSC

To monitor neurospheres formation of shPDGFRα-CSC *vs* control cells (pLKO.1), cells are dissociated into single cells and 1x10^4^ cells/well were plated in triplicate on 6-well dishes. Cells are imaged in phase-contrast after 3 and 7 day in growth medium using an Olympus CX41 microscope with 20X/10X objectives. For cell differentiation, cells were dissociated in single cells and plated onto matrigel-coated 100 mm dishes in presence of 5% fetal calf serum (FCS) for 1, 2 and 4 days. To assess the effects of pharmacological treatments, time course experiments were conducted on cells undergone to growth factors withdrawal for 2 days and then stimulated with exogenous PDGF-AA (Peprotech) alone or combined with 10μM of Crenolanib (CP-868,596; Selleckchem), for different time points (5-10-30-120 minutes until 24 hours).

### RT-qPCR assay

Total RNA was extracted using Triazol and by RNeasy mini kit (Qiagen, USA). cDNAs were obtained using QuantiTect Reverse Transcription kit (Qiagen, USA). Quantitative Real Time Reverse Transcriptase PCR (RT-qPCR) was conducted in triplicate using SYBR Hi-ROX kit (Bioline, UK). RT-qPCR was performed with a 7900HT instrument equipped with SDS2.2 software (Applied Biosystems, CA). The sequences of oligonucleotides used for RT-qPCR were described in Table 1. The Fold change (Fc) were obtained by normalization of RNAs expression respect to selected housekeeper genes (GAPDH, TBP) and respect to pLKO.1 cells.

**Table 1:** List of primers for RT-PCR analysis

Gene ID	Name		Sequences 5′→3′
NM_001128128.2	hZEB1	For	ATGACCTGCCAACAGACCAG
		Rev	TTGCCCTTCCTTTCCTGTGT
NM_001171653.1	hZEB2	For	AAGCCTCTGTAGATGGTCCAG
		Rev	GTCACTGCGCTGAAGGTACT
NM_003380.3	hVIM	For	AGAGGAAGCCGAAAACACCC
		Rev	TCAAGGTCAAGACGTGCCAG
NM_000474.3	hTWIST	For	TCAAGAGGTCGTGCCAATCA
		Rev	ATGGTTTTGCAGGCCAGTTT
NM_001166055.1	hEDNRA	For	GAGGTTTTCTGAAGCCGGGG
		Rev	TGTGCTGCTTTTACACCTTCAC
NM_000115.3	hEDNRB	For	TGCTTGCTTCATCCCGTTCA
		Rev	GGCCAATGGCAAGCAGAAAT
NM_001168319.1	hEDN1	For	TGAGAGGAAGAAAAATCAGAAGA
		Rev	TTTCTCATGGTCTCCGACCT
NM_001306132.1	hFN1	For	TGACAAGCAGACCAGCTCAG
		Rev	CTGTCACACGAGCCCTTCTT
NM_001795.3	hCDH5	For	ATGCGGCTAGGCATAGCATT
		Rev	TGTGACTCGGAAGAACTGGC
NM_004360.3	hCDH1	For	CGAGAGCTACACGTTCACGG
		Rev	CTTTGTCGACCGGTGCAATC
NM_000442.4	hPECAM1	For	TGATGCCGTGGAAAGCAGAT
		Rev	GCATCTGGCCTTGCTGTCTA
NM_002253.2	hVEGFR2	For	ACAGCAACTTGCAGGACAGT
		Rev	GAGCTCGATGCTCACTGTGT
NM_003106.3	hSOX2	For	AATAGCATGGCGAGCGGGG
		Rev	CCGTTCATGTGCGCGTAACT
NM_001173531.2	hOCT4	For	CTCGAGAAGGATGTGGTCCG
		Rev	TAGTCGCTGCTTGATCGCTT
NM_000321.2	hRB1	For	CTCACCTCCCATGTTGCTCA
		Rev	GGGTGTTCGAGGTGAACCAT
NM_002737.2	hPRKCA	For	ATATGTCAACGGTGGGGACC
		Rev	TCCGATGGAAATCTCTGCCG
NM_002738.6	hPRKCB1	For	TCCAGCCCCCTTATAAGCCA
		Rev	GATGGCGGGTGAAAAATCGG
NM_033023.4	hPDGFA	For	CGGATACCTCGCCCATGTTC
		Rev	CGGATGCTGTGGATCTGACT
NM_033016.2	hPDGFB	For	GGTGAGATGTTTATCATGGGCCT
		Rev	CGAGTGGTCACTCAGCATCT
NM_016205.2	hPDGFC	For	TCTGAACCAGGGTTCTGCATC
		Rev	AAGGGGGTAGCACTGAAGGA
NM_025208.4	hPDGFD	For	TCGGTATCGAGGCAGGTCAT
		Rev	GGGAGTGCAACTGTAACGCT
NM_031847.2	hMAP2	For	GCTCTGGCTCCCAGTGTATT
		Rev	GAGCGCTTTTCTGGGCTCTT
NM_001172085.1	hTBP	For	GAACATCATGGATCAGAACAACA
		Rev	ATAGGGATTCCGGGAGTCAT
NM_001256799.2	hGAPDH	For	AGCCACATCGCTCAGACA
		Rev	GCCCAATACGACCAAATCC

### Cell invasion assay

For the invasion assay, 1x10^5^ cells resuspended in 0.3 ml of Stem Medium and placed in triplicate into the top chamber of 150μl matrigel-coated transwell insert (Millipore). The bottom wells contained 0.4 ml of stem medium as control or stem medium with PDGF-AA or EGF (50 ng/ml) as chemoattractant. After 48h, cells on the top surface of the filter are removed with a cottonswab. Thereafter, the filters are fixed and stained with 0.5% of crystal violet and subsequently washed to collect the staining solution. OD values, proportional to the number of cells, are measured by a plate reader at 590 nm (Bio-Rad). These experiments were performed twice and each time in triplicate. The absorbance values were calculated as mean ± SD (n=3) of two different experiments.

### Statistical analysis

Statistical analysis was performed with Prism5 (GraphPad) and Microsoft Office Excel 2013. All data shown are representative of results obtained from two independent experiments conducted in triplicate. The results were analyzed by Two-way ANOVA and Bonferroni's post tests. Data are expressed as mean ± SD (n=3) of 2 independent experiments, and P values ≤0.05 (*), ≤0.01(**), ≤0.001 (***) were considered statistically significant.

## SUPPLEMENTARY MATERIAL FIGURE


