# Correction: PDGFRα depletion attenuates glioblastoma stem cells features by modulation of STAT3, RB1 and multiple oncogenic signals

**DOI:** 10.18632/oncotarget.18268

**Published:** 2017-05-29

**Authors:** Carlo Cenciarelli, Hany E. Marei, Armando Felsani, Patrizia Casalbore, Gigliola Sica, Maria Ausiliatrice Puglisi, Angus J.M. Cameron, Alessandro Olivi, Annunziato Mangiola

**Present:** Reference and callout information were presented incorrectly for reference 24.

**Correct:** The proper reference and callout information are listed below.

**RESULTS**

Cancer stem cells from GBM were isolated as described previously [24]. We were able to collect either core- (c-CSC) or peritumor tissue-derived cancer stem cells (p-CSC) from several primary GBM samples. The two types of CSC form intracranial tumors in immunocompromised mice. Nevertheless, c-CSC showed a higher tumor initiating ability when compared with p-CSC which had a lower tumorigenicity [24].

**REFERENCES**

24. Lama G, Mangiola A, Proietti G, Colabianchi A, Angelucci C, D'Alessio A, De Bonis P, Geloso MC, Lauriola L, Binda E, Biamonte F, Giuffrida MG, Vescovi A, Sica G. Progenitor/Stem Cell Markers in Brain Adjacent to Glioblastoma: GD3 Ganglioside and NG2 Proteoglycan Expression. J Neuropathol Exp Neurol. 2016; 75:134-47.

Original article: Oncotarget. 2016; 7:53047-53063. doi: 10.18632/oncotarget.10132

