# Supplementary material for: PDGFRα depletion attenuates glioblastoma stem cells features by modulation of STAT3, RB1 and multiple oncogenic signals
Source: Oncotarget. 2016 Jun 17;7(33):53047–63. doi: 10.18632/oncotarget.10132 (PMC5288168; doi:10.18632/oncotarget.10132)
Supplement: Supplementary file 1 [file oncotarget-07-53047-s001.pdf]

PDGFRα depletion attenuates glioblastoma stem cells features by modulation of STAT3, RB1 and multiple oncogenic signals

SUPPLEMENTARY FIGURE

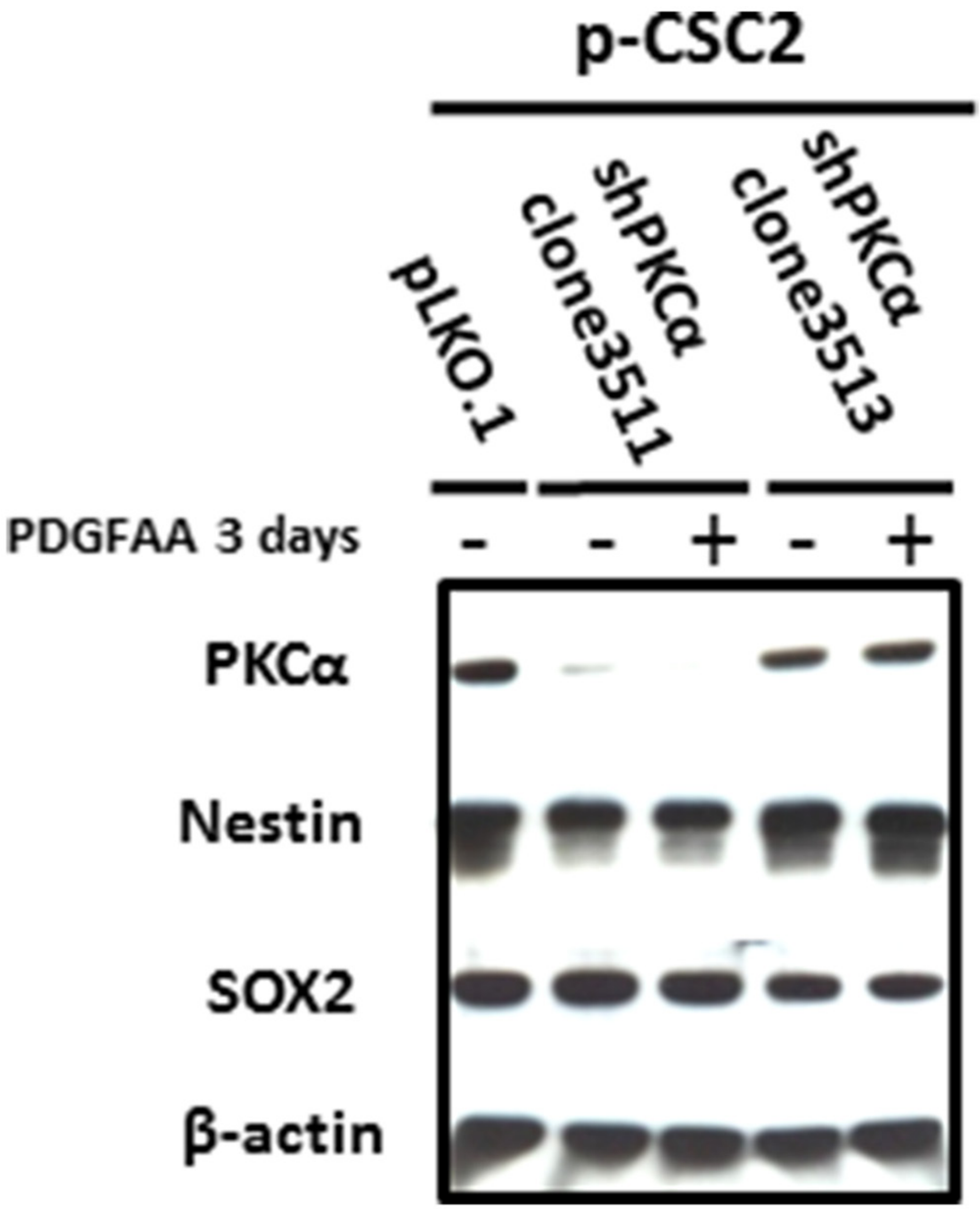

Supplementary Figure S1: PKCα is not involved in the regulation of stemness in p-CSC2. Reduction of PKCα protein expression in shPKCα cell clones does not affect expression of multipotency stem cell markers Nestin and Sox2 compared to pLKO.1 cell, after 3 days of PDGF-AA treatment.
